# Skin Microbiome Analysis for Forensic Human Identification: What Do We Know So Far?

**DOI:** 10.3390/microorganisms8060873

**Published:** 2020-06-09

**Authors:** Pamela Tozzo, Gabriella D’Angiolella, Paola Brun, Ignazio Castagliuolo, Sarah Gino, Luciana Caenazzo

**Affiliations:** 1 Department of Molecular Medicine, Laboratory of Forensic Genetics, University of Padova, 35121 Padova, Italy; luciana.caenazzo@unipd.it; 2 Department of Cardiac, Thoracic, Vascular Sciences and Public Health, University of Padova, 35121 Padova, Italy; dangiolellagabriella@gmail.com; 3 Department of Molecular Medicine, Section of Microbiology, University of Padova, 35121 Padova, Italy; paola.brun.1@unipd.it (P.B.); ignazio.castagliuolo@unipd.it (I.C.); 4 Department of Health Sciences, University of Piemonte Orientale, 28100 Novara, Italy; sarah.gino@uniupo.it

**Keywords:** skin microbiome, collection methods of skin microbiome, diversity of skin microbiome, forensic sciences, human identification

## Abstract

Microbiome research is a highly transdisciplinary field with a wide range of applications and methods for studying it, involving different computational approaches and models. The fact that different people host radically different microbiota highlights forensic perspectives in understanding what leads to this variation and what regulates it, in order to effectively use microbes as forensic evidence. This narrative review provides an overview of some of the main scientific works so far produced, focusing on the potentiality of using skin microbiome profiling for human identification in forensics. This review was performed following the Preferred Reporting Items for Systematic Reviews and Meta-Analyses (PRISMA) guidelines. The examined literature clearly ascertains that skin microbial communities, although personalized, vary systematically across body sites and time, with intrapersonal differences over time smaller than interpersonal ones, showing such a high degree of spatial and temporal variability that the degree and nature of this variability can constitute in itself an important parameter useful in distinguishing individuals from one another. Even making the effort to organically synthesize all results achieved until now, it is quite evident that these results are still the pieces of a puzzle, which is not yet complete.

## 1. Introduction

The mistaken belief that the human body hosts microbes that outnumber our somatic and germ cells by an estimated 10-fold led to an increasing interest in characterizing human microbiota—namely, all the bacteria inhabiting the human body in health or disease [1]—to better understand the balance between human and microbial components, predominantly bacteria, with fungi, viruses, and protists [2]. However, recently, it was demonstrated that the microbial cells which colonize the human body (i.e., microbiota) are at least as abundant as our somatic ones, with a more realistic estimated ratio of bacteria to human cells of about 1.3 [3]. Considering that the human body harbors 500–1000 different species of bacteria and each bacterial strain has a genome containing thousands of genes, it is clear that the total DNA content of microbes inhabiting our bodies, which is the microbiome [1], offers much more genetic diversity than the human genome [4,5]. The fact that different people host radically different microbiota highlights forensic perspectives in understanding what leads to this variation and what regulates it, in order to effectively use microbes as forensic evidence. Therefore, while, at first, microbial forensics was defined as the “discipline of applying scientific methods for the analysis of evidence from a bioterrorism attack, bio-crime, hoax, or inadvertent release of a biological agent or toxin, with attribution as the ultimate goal” [6], subsequently, an expanded definition was proposed as the “discipline of characterizing microbiological evidence to develop investigative leads in criminal and civil cases” [7].

In recent years, there were rapid advances in molecular sequencing and computational techniques. The introduction of massive parallel sequencing (MPS) technology, also referred to as next-generation sequencing (NGS) or high-throughput sequencing (HTS), incredibly improved the amount of sequencing data that is also available for forensic analysis [8]. At the same time, these technologies reduced not only the analytical costs associated with the generation of sequencing data, but also the time of analysis performance. Using NGS to sequence total DNA extracts from any sample allows both the sequencing of the whole genome of a given microorganism and the examination of whole communities of microbes, with the possibility of rapidly and efficiently identifying all different bacterial taxa and strains, thereby obtaining an overview of the resident microbial population [9,10].

Microbiome research is a highly transdisciplinary field with a wide range of applications and methods for studying it, involving different computational approaches and models [11].

The advent of metagenomics, in fact, makes possible the characterization of hundreds of thousands of microorganisms that compose the microbial community of an individual [5], even if these microbial species are difficult or impossible to culture in vitro.

Since it was shown that microbiota variation between different individuals is higher than within the same individual, and that humans have their personalized microbiomes with a large degree of interpersonal diversity, studies focused on the possibility of determining if these differences are great enough to differentiate individuals [12]. In this new research field, skin microbiome analysis appears promising because the skin, as the largest organ of the human body, is a complex living ecosystem that harbors diverse microbial communities at different sites with unique niches, characterized by dry, moist, and sebaceous skin [13,14,15,16,17].

The degree of skin microbiome diversity depends on the taxonomic depth of the analysis. Therefore, while it is possible to identify only a few distinctive taxa amongst various people by considering the phylum of the bacteria, the discriminatory power becomes similar to a fingerprint if the identification is achieved at the genus, species, and strain population levels, with an extremely specific diversity for each person [8,14,15,16,17,18].

The particular composition of human microbial communities is influenced by many factors, such as environment [19,20], development, presence, or absence of diseases [21], habits [22], relationships [12], nutrition [23], and health status in general. Additionally, a bidirectional influence was shown; while environmental bacteria can alter the microbiomes of people who spend their time in there, humans shed bacteria from their body surface into the surrounding environment, changing its bacterial composition [24,25]. Many studies demonstrated how human microbial signatures can be recovered in several indoor or outdoor settings, such as houses [19,26], offices [27], healthcare facilities [28], classrooms [29], dormitory rooms [30,31], restrooms [32], and subways [33].

Given these premises, microbial analysis may allow for the differentiation of humans by the bacteria they harbor, and it can contribute to human identification, while also providing alternative and sensitive targets of genetic investigation. When a person touches an item, a special bacterial signature can be transferred to surfaces, following contact [29,34]. The characterization of these microbial traces of human contact, even by utilizing the same general tools already used for human DNA analysis, allows for the association of individuals with objects and places [35,36,37]. Furthermore, since the composition of a microbiome can provide information about the host’s lifestyle [38], microbiome-based analysis can be used for forensic purposes, such as identifying likely suspects who can be linked to a crime scene [39]. The possibility of matching these microbial profiles to the individual from whom they likely originated highlights similarities between human skin microbiome and friction-ridge patterns analysis or DNA short tandem repeat (STR) typing. In this context, microbial analysis could be more informative than fingerprint analysis when this procedure cannot be carried out due to the presence of heavily smudged prints and substantially partial prints, and it can complement DNA STR typing when only low-biomass or degraded samples are available and it is not possible to obtain a full human STR profile [40].

This narrative review provides an overview of some of the main scientific works so far produced, mainly focusing on the potentiality of using skin microbiome profiling for human identification in a forensic setting.

## 2. Materials and Methods

This review was performed following the Preferred Reporting Items for Systematic Reviews and Meta-Analyses (PRISMA) guidelines [41].

The articles identified in the present review were selected from PubMed and Scopus databases; for the search strategy, we decided to use the following keywords: “microbiota [and] forensic”, “bacteria [and] forensic identification”, and “microbiome [and] human identification”. We used the keywords isolated or combined. We searched for more studies among the reference lists of the selected papers and systematic reviews. In this way, we identified a total of 2965 works on PubMed and Scopus databases.

Three of the reviewers (P.T., G.D.A., and P.B.) carried out the initial search of the papers. They used the protocol of search previously described to identify literature. In the case of disagreements, the consensus of the research supervisors (L.C., S.G., I.C.) was asked. The researchers used the following research order: titles were screened first, then abstracts, and lastly full papers. A paper was considered potentially relevant and its full text reviewed if, following discussion between the two independent reviewers, it could not be unequivocally excluded on the basis of its title and abstract. The full text of all papers not excluded on the basis of abstract or title was evaluated.

Duplicates were removed and a total of 845 works were screened on the basis of the following inclusion criteria: (1) abstract and full text in English language; (2) titles and/or abstracts suggested a personal identification performed by skin microbiome analysis; (3) titles and/or abstracts suggested skin microbial analysis on touched objects; (4) titles and/or abstracts suggested an evaluation of ecological features like skin microbiome’s temporal and spatial stability.

A total of 157 article abstracts were considered during this phase of abstract screening, after which 83 articles were examined in full text for eligibility.

In the end, after full-text examination, we selected 18 experimental studies published between 2009 and 2020 for qualitative synthesis.

To estimate any potential bias that was most relevant for the study, we used the Cochrane tool for assessing risk of bias in randomized trials (RoB 2 tool) and the Risk of Bias in Non-randomized Studies of Interventions (ROBINS-I). All articles included were considered as having a low risk of bias.

**Figure 1:**
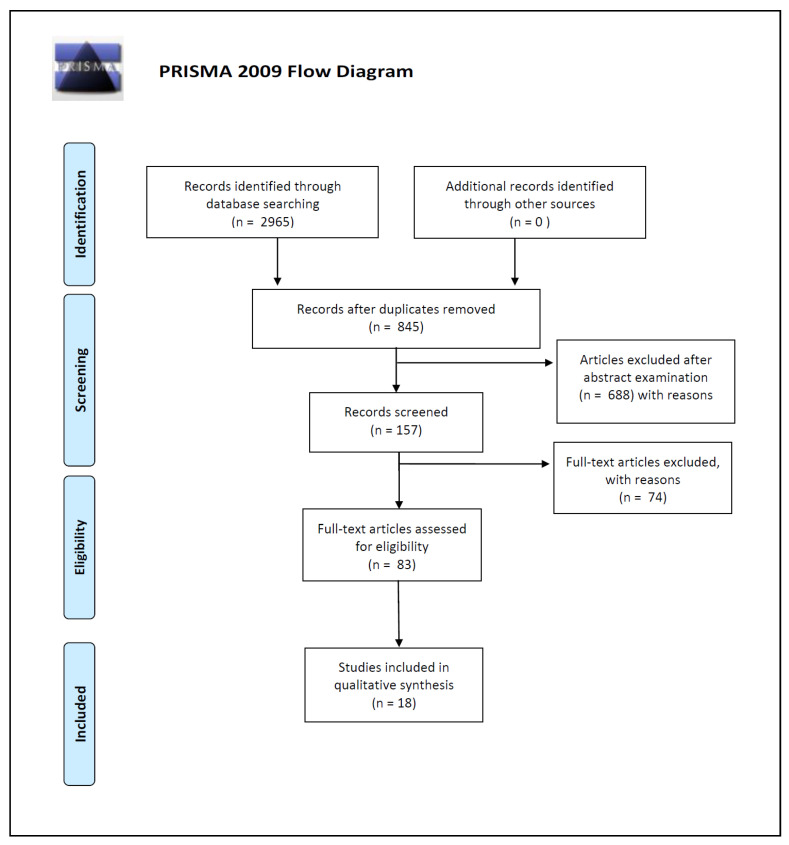
Preferred Reporting Items for Systematic Reviews and Meta-Analyses (PRISMA) 2009 flow diagram.

The number of articles excluded or included were registered and reported in a PRISMA flowchart (Figure 1).

## 3. Results

In order to make the results obtained in the current review more comprehensible to the reader, we summarized selected studies by grouping them into the following three categories: (a) the search for the best identification methodologies; (b) indirect identification: the possibility of linking people to objects and individuals by skin microbial transferring; (c) ecological features: microbiome temporal and spatial stability.

### 3.1. The Search for the Best Identification Methodologies

Historically, microbiology was almost entirely culture-dependent. Therefore, early studies of the human microbiome involved the culturing of the microbes. Since many skin bacteria are difficult to grow, prior to the advent of NGS technologies, only a limited set of information about skin microbiome was available. Currently, there are two approaches that are most frequently used in skin microbiome research. The first one is amplicon sequencing, which works well for samples contaminated by host DNA, such as tissue and low-biomass samples [42], and which relies on sequencing of taxonomic marker genes (usually 16S ribosomal RNA (rRNA) gene for bacteria and archaea and the internal transcribed spacer (ITS) for fungi) after initial targeted PCR amplification. The second one is metagenomic shotgun sequencing, which simultaneously captures all genetic material, providing sequence information on a randomly picked set of DNA fragments extracted from the sample.

#### 3.1.1. The Amplicon Sequencing Approach

The 16S rRNA gene is common among prokaryotes, and it is essential for bacterial life, but it is not present in eukaryotes. This gene, which encodes the small subunit of the bacterial ribosome, is characterized by species-specific variable regions, which are useful for identifying phylogenetic relationships. After sequencing, 16S regions may be analyzed bioinformatically. Highly similar sequences are grouped into operational taxonomic units (OTUs) with an allowed typical degree of sequence divergence of 95%, 97%, or 99%. Since named species genomes are often unavailable for particular marker sequences, OTUs take the place of “species” in many microbiome diversity analyses. The assignment of sequences to OTUs is referred to as binning, and it can be performed by (a) unsupervised clustering of similar sequences, (b) phylogenetic models incorporating mutation rates and evolutionary relationships, or (c) supervised methods that directly assign sequences to taxonomic bins based on labeled training data [43]. The community can be described in terms of which OTUs are present, their relative abundance, and/or their phylogenetic relationships. In general, microbiome differences are assessed by comparing alpha and beta diversity metrics. Alpha diversity metrics quantify within-sample diversity and can be compared across groups. Between-sample diversity can be calculated by beta-diversity measures and is often obtained by comparing feature dissimilarity, generating a distance matrix between all pairs of samples [44]. These distance matrices can be explored by principal coordinate analysis (PCoA), assigning locations to individual samples within a low-dimensional space (visualized as a three-dimensional (3D) graph), so that bacterial community similarities/dissimilarities and variation may be explored.

#### 3.1.2. Shotgun Metagenomics Approach

This is the method of sequencing all microbial genomes within a sample. The information obtained with this alternate method can be used similarly to amplicon sequence-based methods to identify which taxa are present and the relative abundance of each, or to identify gene coding sequences and analyze the functional potential of the microbial community. It is also possible to compare shotgun metagenomics data to reference databases in order to assign taxonomy. With this approach, the possibility of detecting species or strain-specific markers is greatly reduced because the larger the genome(s) characterized, the less read depth would be obtained for any particular site.

In this section, we propose a descriptive analysis of the reviewed studies that focused on evaluating different methodologies of skin microbial-based human identification.

Franzosa et al. in 2015 [45] developed a hitting set-based coding algorithm and applied it to the Human Microbiome Project (HMP) population in order to evaluate whether intraindividual microbial variation is sufficient to allow individual identification within a large population, and whether microbial communities are stable over time. The authors constructed metagenomic codes based on skin, gut, oral, and vaginal microbiome from sets of individual-specific and maximally stable metagenomic features. They verified that microbiome features were generally less unique and less stable than features of the human genome. Nevertheless, codes capturing strain variation in clade-specific marker genes allowed distinguishing a person among hundreds of individuals. In contrast to codes based on the gut microbiome that appeared highly stable, with the possibility of matching the correct person after a time interval ranging from 30 to 364 days, metagenomic features measured at sites on the skin showed a certain instability over time. An alternative method of human identification by microbial forensics was proposed by Schmedes et al. in 2017 [46], where they used supervised learning to attribute skin microbiomes to their donors with a high degree of accuracy. In order to do so, the authors used publicly available shotgun metagenomic datasets from Oh et al. [47], consisting of a wide number of samples of skin microbiomes collected at three different timepoints over a period ranging from five weeks to 30 months from 12 healthy individuals across 17 body sites. After a quality-control consisting of a removal of sequencing adapters, reads with a quality score of less than 20, reads less than 50 bp in length, and any human host-associated reads, a taxonomic classification was performed using a MetaPhIAn2 database, in order to determine the core skin microbial species, which are stable over time and shared by all individuals. Since skin microbial species with these characteristics can be used as forensically relevant targets, Schmedes et al. underlined that *Propionibacterium acnes* was the only species present in all samples at all body sites. Furthermore, as previously reported by Oh et al., this bacterial species has stable and individual-specific strain single-nucleotide variant profiles, and the known *Propionibacterium acnes* pangenome (i.e., the composition of all core and accessory genes present from all known strains of a given species) reaches saturation from all *P. acnes* strains samples across an individual (i.e., all genes from the *Propionibacterium acnes* pangenome are present across all samples).

Taking into account these abovementioned characteristics, Schmedes et al. considered that *Propionibacterium acnes* might constitute a potential forensic target in a supervised learning context. In particular, the authors tried to attribute skin microbiomes to their relative donors, performing two supervised learning methods, specifically, regularized multinomial logistic regression (RMLR) and one-nearest neighbor (1NN) classification, using two feature types derived from skin-microbiome signatures, *Propionibacterium acnes* pangenome gene presence/absence features, and nucleotide diversity of universal clade-specific markers.

By evaluating pangenome presence/absence profiles, RMLR classification accuracy (i.e., the percentage of samples classified correctly) showed a mean of 79.4% (depending on the examined body site, ranging from 66.67% at the ear and interdigital web to 95.24% at the volar forearm), while 1NN accuracies showed a mean of 80.71% (also depending on the examined body site, ranging from 58.33% at the inguinal crease to 96.30% at the hypotenar palm).

By evaluating the nucleotide diversities of universal stable clade-specific markers, shared by all individuals and all time points for each body site, the authors obtained better results, with an RMLR mean accuracy of 87.21% (ranging from 66.67% at the inguinal crease to 100% at the cheek), and a 1NN of 82.2% (ranging from 56.67% at the alar crease to 100% at the inguinal crease and popliteal fossa). These results showed how the nucleotide diversity of stable markers yielded the highest classification accuracies, up to 100% for three body sites (the cheek, the inguinal crease, and the popliteal fossa). The authors also performed an “attribute selection” that allows for the reduction of noise and the elimination of features that do not contribute to the performance of the classifier in order to obtain a comparable classification accuracy even if using a reduced subset of features. They selected an average of 24 markers from 1108 for presence/absence features and an average of 32 markers from 263 clade-specific markers. This feature reduction did not affect classification accuracy, and it resulted in the elimination of potential noise and redundancy in signal. In conclusion, Schmedes et al. also evaluated whether this classification method was robust in time. They assessed that microbial samples collected in a short time interval (five to 10 weeks apart) could be linked to their donors with higher accuracy than microbiome samples collected more than 10 to 30 months apart.

With this work, Schmedes et al. showed how several elements could influence the probability of a correct classification of a given classifier (indeed, it was influenced by feature selection/classifier type and, furthermore, accuracy varied substantially across body sites, and across feature vector type), and they provided further evidence that presence/absence features are less individualizing than nucleotide diversity. Using 1NN classification on nucleotide diversity, Schmedes et al. [46] also observed an unexpected finding; body sites which are likely to be of the greatest forensic relevance, such as the hand (palm), yield highly accurate rates of classification, even if they seem to contain relatively few (approximately 17%) shared phylotypes between the two hands of the same individual, and this body site is often the target of frequent recolonization by daily activities.

In 2018, Schmedes et al. [48], building on their initial work, presented a novel targeted sequencing method, called hidSkinPlex. This sequencing panel was developed based on a candidate marker from Schmedes et al. [46] for skin microbiome profiling for forensic human identification. In particular, it contained 286 bacterial (and phage) family-, genus-, species-, and subspecies-level markers, selected from the MetaPhlAn2 [49] reference database, with >65% of the markers from the dominant skin flora, *Propionibacterium acnes*. The authors initially assessed the performance (i.e., sensitivity and specificity) of the multiplex on three bacterial control-sample panels (i.e., *Propionibacterium acnes*, *Propionibacterium granulosum*, and *Rothia dentocariosa*) by determining the proportion of true positives, false positives, true negatives, and false negatives based on expected marker presence/absence. The multiplex was further evaluated for prediction purposes using microbiome samples collected in eight individuals from three skin sites, the toe web/ball of the foot (Fb), the palm of the non-dominant hand (Hp), and the manubrium (Mb). The choice of two of these body sites (Mb and Hp) was influenced by both their forensic relevance and the possibility of overlapping with sites, which generally show higher classification accuracies, as previously tested by Schmedes et al. [46]. The third body site (Fb) was chosen to test the potential of discriminating among individuals through skin-foot microbial communities by using targeted enrichment of informative hidSkinPlex markers. The authors used supervised learning, specifically, RMLR and 1NN classification, to attempt to attribute skin microbiomes to their hosts, using “leave-one-out cross-validation” (LOOCV) as a training set. Hp samples showed the highest classification accuracies, ranging from 95.8% to 100%, while classification accuracies for Mb ranged from 70.8% to 95.8%. These classification accuracies turned out not to be significantly different from those previously calculated by Schmedes et al. [46] using shotgun data. The use of enriched hidSkinPlex markers allowed for greater amplification of common markers shared by all individuals on Fb, obtaining largely different results from using shotgun sequencing data instead (23%). Classification accuracies for the Fb ranged from 54.2% to 83.3% and increased up to 91.7% using non-universal markers at each threshold. The authors achieved a high degree of accuracy (up to 97.2%) in classifying all 72 samples, independently from the body site of origin, and they also succeeded in predicting body site origin in up to 86% of cases. Finally, Schmedes et al. tested if skin microbiome profiling could be used in conjunction with low-biomass or degraded samples which fail to yield full human STR/single-nucleotide polymorphism (SNP). In order to do so, the authors generated human short tandem repeat and single-nucleotide polymorphism profiles (Illumina ForenSeq panel A) from a skin swab (Fb, Hp, and Mb) of a single female subject. Only few samples swabbed from Fb and Mb yielded complete or nearly complete STR/SNP profiles (92–99%). All samples from the hand generated partial profiles, ranging from 32% to 52%, with the lowest number of alleles detected. However, the analysis of these same samples with hidSkinPlex showed a classification accuracy of 100%, underlining the potential of skin-microbiome profiling for investigative purposes, especially when it is not possible to generate complete human DNA profiles from low-biomass samples.

In work published in 2019, Woerner et al. [50] compared two important classification strategies for human identification by microbial analysis. They examined a measure of ecological composition (the nucleotide diversities of universal stable clade-specific markers) to highlight differences in classification accuracy with a measure of genetic relatedness (the patristic distance from a phylogenetic tree). The comparison was based on the analysis of the targeted sequencing of 286 markers from 22 microbial taxa, sampled in 51 individuals across three body sites (already examined by Schmedes et al. [48]) measured in triplicate (the manubrium—Mb, the palm of the hand—Hp, and the ball of the foot—Fb). The nearest neighbor (NN) and reverse nearest neighbor (rNN) classifiers were constructed based on the pooled data to predict the human host, either based on the Euclidean distance of the nucleotide diversities of universal stable clade-specific markers or using the phylogenetic distance on the tree. This study showed that, considering classifier accuracy as a function of NN and rNN distance, the proportion of correctly classified samples was consistently greater using the Euclidean distance on nucleotide diversity (reaching accuracy of 71% and 78%, respectively) than the proportion obtained with the patristic distance approach (54% and 63% accuracy, respectively). Therefore, the authors concluded by assessing that approaches which use similarity in nucleotide diversity perform better than those that are based on a phylogenetic distance. Woerner et al. also presented the results of a case study of a single misclassified point examined by Wright’s index of fixation (FST), i.e., a genetic distance (on a 0–1 scale) between populations, which can be estimated by comparing the number of pairwise differences within populations with the number of pairwise differences between populations, constituting a hybrid of the abovementioned approaches. They noticed that FST, especially extreme values in the distribution of FST, was more indicative of inter-individual differentiation than intra-individual differentiation, and they suggested investigating future classifier strategies, which include the selection of high FST markers. Furthermore, since this approach is similar to how ancestry informative markers are often selected in human population studies, they hypothesized that the methods already available for predicting human populations may be extendable to the microbiome and appropriate for differentiating human hosts from their microbial communities.

### 3.2. Indirect Identification: the Possibility to Link People to Objects and Individuals by Skin Microbial Transferring

#### 3.2.1. Keyboards and Computer Mice

In 2010, Fierer et al. [35] firstly demonstrated the potential of microbiome analysis for forensic identification. They showed how the analysis of the skin microbiome could be used to link an individual to an object they touched, illustrating that it is possible to link touched surfaces to individuals who touched the objects by comparing the bacterial communities yielded by an individual’s skin and an object’s surface. In particular, they performed three interrelated studies:

- the first one (the “keyboard study”) aimed to compare bacterial communities on the keys of three personal computers to the communities yielded by the fingertips of keyboard owners;
- the second one (the “storage study”) concerned the evaluation of long-term temporal stability of bacteria yielded by skin on swabs, which were stored at -20 °C or left under typical indoor environmental conditions (about 20 °C) for up to 14 days;
- the last study (the “computer mouse study”) analyzed the possibility of linking objects to specific individuals by comparing bacterial communities found on their computer mice against a database filled with information derived from the hand of the owner and from more than 270 hands that never touched the mouse.

Results from the “storage study” are shown in the paragraph about “Ecological features: microbiome temporal and spatial stability”. For the “keyboard study”, Fierer and colleagues swabbed both individual keys of three personal computer keyboards (25–30 keys per keyboard) and the fingertips of the owner and nearly exclusive user of each keyboard. The keyboards were last touched more than 30 min before sampling. Space bar keys from 15 other private and public computer keyboards were also swabbed in order to compare the bacterial communities on the three keyboards to other miscellaneous keyboards. All swabs were stored at -80 °C for less than one week and then DNA was extracted. A barcoded pyrosequencing procedure was used to determine bacterial community composition. For the “computer mouse” study, the authors recruited nine adults who worked in the same building. Then, they swabbed the entire exposed surface of each computer mouse and the palm surface of each owner’s dominant hand, which is likely used to move the mouse. Every mouse was last touched by the owner more than 12 h previously. All swabs were stored at -80 °C before DNA extraction. The authors compared bacterial communities yielded on computer mice against a database that was compiled with bacterial communities from the hand of the mouse’s owner and from 270 other hands belonging to healthy male and female volunteers between the ages of 18 and 40 who never touched the mouse.

Regarding the first study, Fierer showed that bacterial communities on the fingertips of the owner of each keyboard are similar to the communities on the owner’s keyboard, and bacterial communities on the fingertips or keyboard of a given individual are far more similar to each other than to fingertips or keyboards from other individuals. Since inter-individual differences in fingertip and keyboard communities exceed the differences between bacterial communities on the fingers and keyboards belonging to a given individual, they affirmed that differences in keyboard-associated communities are likely caused by direct transfer of fingertip bacteria. With the “computer mouse” study, they achieved the important demonstration that bacteria yielded on a personal object are more similar to the owner’s skin bacterial communities than to a general population microbiome, such as that harbored by the examined volunteers. This result was obtained calculating phylogenetic distance with the UniFrac algorithm, which uses the degree of phylogenetic overlap between any pair of communities with points that are close together representing samples with similar bacterial communities. The authors concluded that every person leaves a unique bacterial “fingerprint” on touched surfaces. The bacterial DNA can be recovered from these surfaces and can be linked to the person who touched the surface, transferring their fingertip bacteria. Since, under standard indoor conditions, skin-associated bacterial communities persist on objects up to two weeks after the object was last handled, the object’s microbiome can be analyzed for forensic tools [35]. This kind of analysis might be useful for identifying the object, especially when clear fingerprints cannot be obtained, such as from fabrics and smudged surfaces.

#### 3.2.2. Cell Phones

In 2014, Meadow et al. [51] investigated if hand-associated microbiota could be detected on a phone. Meadow enrolled 17 volunteer participants, who sampled the touch-surfaces of their own mobile phone for approximately 20 s, as well as their own thumb and index finger on their dominant hand (three samples for each of 17 participants).

In order to quantify the shared bacterial communities among phones and fingers, Meadow used two different approaches. In particular, the OTUs turnover was calculated by using the Jaccard taxonomic metric, and the discriminant analysis was conducted by using the Canberra taxonomic metric. The two fingers from each participant shared, on average, 32% of OTUs, while both fingers shared about 22% of OTUs with their respective phones. These percentages increased notably when Meadow limited the analysis to only those OTUs which represented more than 0.1% of a single person’s dataset, obtaining that, on average, 82% of OTUs were in common between index fingers and phones, while 96% of OTUs were shared between index fingers and thumbs. Women volunteers, considered as a group, showed a stronger microbiological connection to their phones than men ones. In fact, the bacterial community composition on women index fingers was not significantly different from that sampled on their mobile phones (*p* = 0.327). Otherwise, there was a significant difference for men (*p* = 0.001). However, when considered on an individual basis, by comparing each person’s index finger to their own phone and then to their average Jaccard similarity with other people’s phones, people shared more bacterial OTUs with their own phones than with phones belonging to other people.

In 2015, Lax et al. [37] published the results of two studies. The first one, called “time series study”, consisted of a longitudinal sampling of phone microbial communities. Lax and co-workers collected microbial samples from the mobile phone of two individuals every hour over the course of two periods of 12 h on consecutive days. The second study, called “biogeographical study”, consisted of the collection of microbial samples from phones belonging to attendees of three different national/international conferences. They noticed dissimilarities in microbial community composition, making it possible to determine, regarding the first study, the phone’s owner and, regarding the second study, which of the three conferences was attended by the phone’s owner.

#### 3.2.3. Fabrics

In 2016, Lee et al. [52] examined microbial communities raised on fabrics following hand contact. They asked three healthy, adult Korean volunteers to touch one of three different fabrics (100% cotton, 55% cotton–45% polyester fabric, and 100% polyester). They collected samples by swabbing the fingertips and palms of each volunteer and each different fabric’s surface. To obtain a precise comparison, they also swabbed the fabric immediately prior to the contact. Half of the samples were cultivated, and the other half were directly used to extract DNA. They firstly noted that microbiome composition varied markedly between samples, with non-cultured samples showing much more species diversity than cultured ones, where only one species (*Bacillus anthracis* group) was predominant. Lee et al. also described that an individual’s bacterial hand communities showed different prevailing microorganisms that closely match bacterial communities raised on the fabric’s surface they touched. This study allows reconstructing of a correspondence between the bacterial community in touched fabrics and the microbiome of someone’s hand.

#### 3.2.4. Handled Objects in Crime Scenes

Kodama et al. in 2019 [53] tried to characterize the post-mortem skin microbiome from 16 scenes of death in the City and County of Honolulu to test whether objects at the scenes can be linked to individual decedents. They also aimed to demonstrate that the post-mortem skin microbiomes are stable during repeated sampling up to 60 h. In order to do so, they collected different microbial samples by swabbing of objects. In particular, they swabbed up to 10 handheld objects found at 16 death scenes located at the decedent’s residence, swabbing a total of 98 objects, 88 of which yielded sufficient genetic material for sequencing. The most commonly swabbed objects (>5% of samples) included doorknobs/handles, phones, water taps, wallets/purses, lighters, computer devices, remote controls, and medical devices. The authors also swabbed the plastic sheet wrapped around the decedent for morgue transit and storage, as well as the right palm of the decedent, using a dry double-headed cotton swab for 10–15 s on three occasions: (1) between 10 and 45 min after arrival; (2) upon arrival at the morgue; (3) at intervals of 6 h while the remains were stored at approximately 6 °C until autopsy or external examination. Finally, they collected the skin microbial communities of 30 living residents of the same geographical area (island of Oahu). Post-mortem skin microbiomes correctly associated with objects at an average accuracy rate of 75%, but the level of accuracy varied by scene and by object. Seven death scenes were associated with an accuracy rate of 100%, accuracies ≥60% were observed at four death scenes, and the remaining five scenes were associated with an accuracy rate ≤50%. Some objects were associated with an accuracy of 100%, including medical devices (6/6), bottles (4/4), pipes/bongs (4/4), handled objects (2/2), book (1/1), drinking vessel (1/1), eyeglasses (1/1), identification badge (1/1), recliner armrest (1/1), and a steering wheel (1/1). Other objects were associated with an accuracy ≥67%, including computer devices (5/6), remote controls (5/6), phones (8/10), door handles (6/8), and light switches/control panels (2/3). Three objects were associated with an accuracy <60%, including water taps (5/9), wallets/purses (4/8), razors (1/2), and lighters (2/6). The remaining objects (keys, cosmetics, hairbrushes, dumbbell, harmonica, nail clippers, and watch) were not correctly associated (0/11). This important variation in accuracy was explained by Kodama and colleagues. They assessed that the object which was poorly associated could have been touched by different individuals. In fact, objects associated with an accuracy of 100% were typically handled by a single user. The authors also underlined that it is difficult to determine when and if the decedent last handled an object, and this factor can markedly affect association accuracy since microbial communities can change over time, losing their individual characteristics. Some objects may also be made of materials that prevent or inhibit microbial colonization.

#### 3.2.5. Direct and Indirect Contact between Individuals

In a work published in 2020, Neckovic et al. [54] investigated the possibility of skin-microbiome transferal between individuals in a controlled setting. Neckovic evaluated whether skin microbiome could be transferred through direct, skin-to-skin contact, or indirect contact via various substrates, including papers, cotton fabrics, glass marbles, and hands. In order to do so, the authors enrolled six participants, who were placed into three pairs in which they remained throughout the study. Neckovic investigated two modes of transfer, which were called “Mode 1” and “Mode 2”. Mode 1 was composed of two parts; Mode 1.1 involved the transfer of right-hand microbiomes through a vigorous, 30-s handshake between participants and Mode 1.2 involved transfer from the right hand after the handshake onto a single substrate, which was rubbed (paper and cotton fabric swatches) or rolled (glass marble) in the right hand for an additional 30 s. The left half of each individual’s right hand (palmar side) was swabbed following Mode 1.1, to ascertain that the handshake caused the transfer of the pair participant’s hand-associated microbiomes. Following Mode 1.2, which involved the transfer onto the substrate, microbial samples from the substrates were collected. Mode 2 involved the investigation of indirect transfer of hand-associated microbiomes. It involved the transfer of left-hand microbiomes onto a single substrate for 30s, followed by participant pairs swapping cotton or paper swatches or glass marbles and repeating the process for an additional 30 s. The results from pairwise permutational multivariate analysis of variance (PERMANOVA) analyses revealed a distinct clustering of participant pairs, based on Jaccard and unweighted Unifrac distances between samples. Therefore, the authors inferred that, as previously demonstrated [35,37,51], individuals may be distinguished using unweighted Unifrac distances of skin-associated microbiomes and the percentage of shared OTUs using Jaccard similarities. Furthermore, Neckovic demonstrated the transfer of hand-associated microbiomes across all involved participants within each participant pair and substrate types, regardless of the direct or indirect mode of transfer.

### 3.3. Ecological Features: Microbiome’s Temporal and Spatial Stability

It remains unclear how long microbial fingerprints can last unchanged on a host’s skin and surfaces, making it possible to analyze these traces and obtain reliable results. It was demonstrated, in fact, that skin microbiota shed by an individual can change over time, undergoing degradation within hours. Furthermore, a temporal variation was observed in human skin microbial composition. These factors can constitute a great limit to microbiome-based methods of human identification.

#### 3.3.1. Microbiome and Identification of Living People

Before tackling the topic of the stability of the microbial traces that can be collected on touched objects or environments (as microbes are no longer harbored on the host’s skin), it is useful to illustrate some of the studies related to changes in skin microbiome over time. In fact, variation in human skin microbial composition could compromise the forensically interesting possibility of connecting crime-scene objects to subjects who touched them if crime suspects are detected after a long period of time.

In 2009, Grice et al. [16] characterized the skin microbiome’s temporal variation. They selected 20 skin sites, representative of distinct niches, and they collected follow-up samples after four to six months from five healthy individuals. The microbial composition in the external auditory canal, the inguinal crease, the alar crease, and the nares were temporally stable. In contrast, the microbiome of the popliteal fossa, volar forearm, and buttock showed appreciable variation between the two sampling times. The authors, therefore, inferred that the longitudinal stability of skin microbial communities is site-dependent. They also assessed that bacterial composition, according to OTUs-based analysis, was similar across all individuals in nares and backs, while interdigital web spaces, toe webs, axillae, and umbilici showed a high degree of interpersonal variation, appearing to be the least similar sites.

Similar results were published by Costello and co-workers, who analyzed 27 body sites, including 18 skin sites, in healthy adult volunteers, four times at day 0, day 1, day 90, and day 91 [15]. They highlighted a high degree of spatial and temporal variability. In fact, only up to 12% of the phylotypes were detected at all sampling times for each person. In their study, the skin microbiome showed higher temporal diversity than the gut and the oral ones. The authors concluded that skin microbial communities, although personalized, vary systematically across body sites and time, with intrapersonal differences over time being smaller than interpersonal differences within all habitats examined.

Microbial temporal variability was defined as a personalized feature of the human microbiome by Flores et al. [55], who investigated the temporal dynamics of the forehead, gut (feces), palm, and tongue microbial communities of 85 college-age adults from three USA universities in order to provide a more complete picture of the range of normal variability of the human microbiome. The authors quantified the amount of temporal variability in diversity of each body habitat, by calculating the coefficient of variation for three alpha-diversity metrics (phylogenetic diversity, phylotype richness, and Shannon index) for each individual. The authors observed that individuals differed in the degree of temporal variability and established that the degree and nature of variability constituted in itself an important parameter useful in distinguishing individuals from one another. They also tried to identify factors eventually associated with this variation, such as antibiotic consumption. Flores et al. observed that individuals who took antibiotics over the time of the experiment did not show an increased temporal variability in microbial communities.

A longer time interval was examined by Oh et al. [47], who collected over long (1–2 years) and short (1–2 months) time intervals at three time points a total of 594 samples from 12 healthy individuals across 17 skin sites in order to assess the effect of time on microbial communities. The authors, based on analyses at the strain and single nucleotide level, surmised that, in the absence of major perturbations, including extrinsic and intrinsic conditions, such as antimicrobial treatment, probiotics, prebiotics, long-term environmental relocations, diet, immunosuppression, illness, the dominant characteristics of skin microbial communities would remain stable indefinitely because this stability was determined by the maintenance of strains over time rather than by the reacquisition of common species from the environment to skin. Therefore, despite the continuous perturbation that human skin undergoes in daily life, healthy adults stably maintain their skin communities for up to two years.

Partially in contrast to previous findings, in a recent study performed on full-thickness skin biopsy specimens collected from 50 participants (24 males and 26 females) by Bay et al. [56], bacterial community composition and overall bacterial richness (as OTUs) did not differ between the two anatomic locations examined (hip and knee). The authors explained this absence of significant differences by underlining that both anatomic sites were dry skin habitats with similar topographical conditions. Additionally, Bay et al. showed a stark contrast between dermal and epidermal bacterial communities and underlined that the variability in skin microbial composition is mostly observed in the epidermal compartment, while the dermal compartment’s community, which basically consisted of a specific subset of the epidermal one, is significantly less variable among individuals.

The temporal stability of microbial communities is also influenced by habits and interpersonal activities. Recently, in 2019, Williams et al. published a study that investigated the possibility of identifying individuals by analyzing their pubic area microbiomes from samples collected over a period of months. They enrolled a total of 43 volunteers, ranging from 25 to 68 years of age, who declared themselves to be sexually active. Volunteers were divided into two groups. The first group included 12 couples that were sex partners. The second group was composed of a random subset of 20 participants, including five of the 12 pairs of couple participants. The first group was asked to collect pubic mound hair and pubic mound swab samples at three different time intervals: week 0, week 6, and week 12. In case the participants did not have pubic mound hair, they were asked to collect pubic mound swabs only. Furthermore, if possible, the sexually active couples were instructed to collect at least two additional samples within 72 h from sexual intercourse, but at approximately two-week intervals from another collection time point. Participants reported various levels of sexual activity in the seven days prior to sample collection, with 10 of 12 couples and 10 individuals without partner participation stating that they were sexually active seven days prior to the collection of at least one sample. The authors, performing hierarchical clustering on the presence/absence matrix for every OTUs in each sample, noticed that couples clustered in proportion to their level of sexual activity. In particular, the two couples who reported not being sexually active during the seven days prior to collection for any of the time points were the only couples whose components systematically clustered separately. Nevertheless, since an increased frequency of sexual activity does not always guarantee increased microbiome similarity, Williams and colleagues concluded that having a single sexual intercourse might not be detected by analyzing microbial transfer. In order to make their analysis more complete, the authors also assessed whether sharing the same environment could influence the possibility of correctly classifying couples. Therefore, after six months, the second group’s participants collected swab samples from sites that were supposed to not be exposed to regular contact, such as the inner elbow and behind each ear. Surprisingly, the analysis of samples from these “hidden sites” showed that couples share the microbial profiles similarly to what takes place in the pubic area. Nevertheless, in contrast to pubic mound microbial communities, in this case, couples were not observed clustering in proportion to their level of sexual activity.

#### 3.3.2. Microbiome and Identification Analysis after Death

Another important field is that concerning microbiome variation after death, with a documented microbial community succession occurring after death in a predictable way, making it possible to provide a regression model for estimating post-mortem interval. However, in a forensic setting, it is fundamental to assess whether these chronological changes occurring after death invalidate the possibility of linking microbial traces collected from an object touched *in vitam* by the dead person to their cadaver.

Pechal et al. [57], hypothesizing that the initial post-mortem microbiome would be a reflection of the host microbiome preceding death, in 2014–2016, collected samples at a single post-mortem time point from 188 cadavers as part of a routine death scene investigation at the Wayne County Medical Examiner’s Office, Detroit, Michigan, USA. Samples were collected from six body sites. This large-scale study showed that, for bodies with an estimated PMI of less than 48 h, microbial diversity appeared stable with distinct microbial communities still inhabiting different body sites. These results established a foundational baseline of post-mortem microbial characterization and suggested that the taxa of the first 24–48 h after death would most represent antemortem microbial communities. Therefore, in a forensic setting, within the first 48 h after death, decedent skin microbes may still be useful for linking them to spaces and objects.

In their study, Kodama et al. [53] observed that post-mortem skin microbial communities remain stable during repeated sampling up to 60 h (i.e., during the mean supposed time leading up to autopsy, after scene processing, morgue transport, and storage) and can be associated with objects at the death scene. The authors collected samples from plastic sheets wrapped around the decedent for morgue transit and storage, as well as the right palm of the decedent using a dry double-headed cotton swab for 10–15 s, three times: (1) between 10 and 45 min after arrival; (2) upon arrival at the morgue; (3) at intervals of 6 h. The remains were stored at approximately 6 °C until autopsy or external examination. The similarity observed between microbiomes from the skin and plastic sheets reflected microbiome stability. Corresponding with the results from the study by Pechal and co-workers [57], they observed that an ante mortem microbiome (that is, one associated with a living person) requires more than 48 h to become a post-mortem one (that is, the microbiome associated with decomposing remains). The skin microbiome samples to be used as trace evidence can, consequently, be collected upon a cadaver’s arrival at the morgue or during autopsy, even if collection at the crime scene is still recommended.

#### 3.3.3. Touch Microbiome

In 2010, Fierer and co-workers [35], in one of their three aforementioned interrelated studies (the “storage study”), swabbed the right armpit of two healthy adult individuals using 16 moistened swabs per individual. Half of these swabs were frozen at −20 °C before DNA extraction; the other half were stored under typical indoor conditions at the laboratory, where the temperature was kept at 20 °C and there was fluorescent light on for about 8 h per day. Bacterial DNA was extracted from four replicate swabs per storage condition after either three days or 14 days, with the DNA stored at −80 °C before analysis. With this “storage study”, the authors demonstrated that storage under above-mentioned typical indoor conditions has little or no influence on bacterial community composition during a maximum time interval of two weeks.

The variation over time in skin microbiota traces under a forensic light was investigated in a work published by Wilkins and colleagues in 2017 [58]. Wilkins et al. studied the possibility of matching individuals to their places of residence, even if a long time passed between skin and surface sampling. Wilkins et al. collected microbiota samples from household surfaces (including bed headboard surfaces, blanket surfaces, fridge-door seal surfaces, kitchen ventilator surfaces, remote control surfaces, shower curtain surfaces, toilet flush button surfaces, and television screen surfaces), household air (bedroom air, kitchen air, living room air, and toilet air), and residents’ skin (forehead skin, left forearm skin, left palm skin, right forearm skin, and right palm skin) in nine Hong Kong residences four times throughout 2014, in winter, spring, summer, and autumn. They determined microbiota composition through the analysis of the 16S rRNA region. The most abundant family across all surface samples was a human skin-associated one, the *Moraxellaceae*, dominated by the skin-colonizing genus *Acinetobacter*. The authors collected other skin-associated families from household surfaces, including *Staphylococcaceae*, *Micrococcaceae*, *Corynebacteriaceae*, and *Streptococcaceae*. These results showed that the major source of household surface microbiota was household occupants’ skin. Furthermore, since greater similarity was observed in the microbial composition of skin samples obtained from cohabiting people, the authors suggested that there is a sort of microbiota exchange, which can be direct, between individuals, or indirect, via common shared reservoir, such as household surfaces. The results also showed more similarities in samples collected from the same residence and season than those from different residences in the same season. Wilkins et al. also noticed that accuracy decreased consistently, in both directions, when skin samples were collected before surface samples, or when skin samples were collected after surface samples. They concluded that changes in microbial trace composition that prevent matching microbial traces with the person who left them, making them lose their forensic potential, occur selectively against low-abundance microorganisms, which are unfortunately the most useful in identifying the individual.

## 4. Discussion

The skin is the largest organ of the human body and it is colonized by millions of microorganisms, whose composition is influenced by many factors, both endogenous, depending on the host (i.e., age, ethnicity, etc.), and exogenous, depending on the environment (i.e., diet, geography, etc.).

Since the skin constitutes the human body’s exterior interface with the environment, it is important in a forensic setting to analyze all skin-related traces, even microbial ones. In recent years, the advancements in NGS technologies exponentially increased the amount of sequencing data available for forensic analysis, creating the possibility, not only to identify and characterize the genes present within a given microbial community, but also to comprehend the dynamics occurring between the taxa comprising the total community. This procedure, rather than replacing current methods used for human identification, could implement these methods when it is not possible to obtain adequate results. The human DNA degradation process can cause a shortening or a fragmentation in DNA molecules resulting in highly degraded samples with partial STR profiles. Most studies focused on the skin bacterial community, with a marked paucity of data on microorganisms other than bacteria. However, bacterial DNA seems to be better protected from environmental conditions (i.e., chemical and physical agents, such as pH changes, ultraviolet (UV) radiation, heat, and humidity) than human DNA and, therefore, more solidly attached by hydrolytic enzymes because of its structure, consisting of a circular molecule of DNA, and its localization into a living prokaryotic cell furnished with a cell wall made up of a peptidoglycan matrix. We owe the first pioneering intuition in the use of the microbiome for human identification to Fierer and his colleagues in 2010 [35]. Since then, several studies were published with the purpose of exploring the possibility of using the skin microbiome to connect people to an object or a place with important repercussions in the forensic field. As described before, we selected 18 works in order to provide an overview of what was experimentally ascertained so far in this recent field of research. All the studies show, albeit with different levels of accuracy, the possibility of linking a person to different touched objects, such as keyboards [35], computer mice [35], cell phones [37], fabrics [52], and different objects collected from indoor crime scenes [53], through microbial analysis.

Since human identification is a comparative analysis, the microbial trace has to be compared with a reference sample constituted of skin microbial communities in order to be linked to the person who left it behind. In the past few years, several studies focused on stability in the human skin microbiome over time. The examined literature ascertained clearly enough that skin microbial communities, although personalized, vary systematically across body sites and time, with intrapersonal differences over time smaller than interpersonal ones, showing such a high degree of spatial and temporal variability that the degree and nature of this variability can constitute in itself an important parameter useful in distinguishing individuals from one another [15,16,47,55]. However, taking into account that most interpersonal differences seem to be confined to the epidermal compartment of the skin [56], the relationship between the dermal and the epidermal community deserves to be more deeply understood. Further studies are needed to determine whether it is effectively possible to individuate a dermal core “universal microbiota”, which is less affected by external factors and, therefore, possibly more stable over time. Subsequently, it should be assessed in what proportion this less individualizing dermal core microbiome can eventually be transferred to the objects, once touched, in order to extrapolate only the information that can be useful in the identification process. Furthermore, there remains to be explored the stability of the microbial profile after the assumption of antibiotic therapies and changes in its composition, structure, and function caused by the occurrence of skin diseases.

The heterogeneity observed in analyzed experimental works makes it impossible to conduct quantitative comparisons across studies. The pronounced differences in test methods and presentation of data often prevent the identification of the contribution of a particular methodological development when more than one component of the approach changes. Moreover, the sample size for most studies is small. If a small-size sample is analyzed, large differences between the sample and the general population can arise simply by chance and it is, therefore, difficult to interpret results and generalize. There is a general lack of real forensic casework. Furthermore, with regard to studies under controlled conditions, it is not yet ascertained which are the best methods for sampling, assessing, or reporting of microbiome data. These general aspects are not undoubtedly defined because of the lack of information concerning differences between the techniques applied until now. In this novel research field, it seems mandatory to identify standard operational protocols for sample collection, handling, preservation, and for data extraction and analysis, with the creation of reference genome databases. So far, only a few studies were expressly designed for method implementation and comparison [45,46,48,50]. It is necessary to generate standards and guidelines to make it possible to consistently achieve the same results using the same methods under the same circumstances, obtaining statistically and scientifically meaningful conclusions. To ensure that the analytical methodology is accurate, specific, and reproducible, validation processes should also be conducted. The accurate interpretation of the obtained results depends on the development of bioinformatics processing and data analysis and the implementation of updated DNA databases.

The possibility of using the microbiome in a forensic context is strictly connected to the stability of the microbial trace to be analyzed. The persistence on inanimate objects under typical indoor condition of microbial traces was evaluated in 2010 by Fierer et al. [35], who showed that microbial communities, collected by swabbing on the armpit of two healthy individuals, remained stable during a maximum time interval of two weeks if swabs were stored in a place where the temperature was kept at 20 °C and where there was fluorescent light on for about eight hours per day. The variation over time in skin microbiota traces was also the subject of a study published by Wilkins and colleagues [58], who observed changings in microbial-trace composition occurring selectively against low-abundance microorganisms, which are the most useful in identifying the individual.

There is an evident lack of studies evaluating how long bacterial DNA persists once deposited on a surface, both under controlled environmental conditions and in real forensic casework conducted in indoor or outdoor settings. More research needs to be carried out to investigate the effect that additional factors have on both short-term and long-term microbiome stability.

Another important research field concerns temporal succession in microbial community composition after death. The ecological changes in the skin microbiome occurring after death could, in fact, prevent re-association between a touched surface and the person who touched it as soon as the skin microbial communities change, following the death of the host. Current findings [53,57] demonstrate that microbes provide an accurate tool for forensic identification up to 60 h after death since, during this time lapse post-mortem, microbial communities quite precisely represent ante-mortem ones. These results have important practical consequences, making it possible to collect skin microbiome samples to also be used as trace evidence upon a cadaver’s arrival at the morgue or during autopsy, if it is known that the time elapsed since death does not exceed 60 h. The aforementioned results, while highlighting the potentiality of microbial analysis in human identification for forensic purposes, with important differences in microbial community composition and function across different people and locations, showed a certain degree of fragmentation. Consequently, even making the effort to organically synthesize all results achieved until now, it is quite evident that the various studies performed so far are still pieces of a puzzle which is not yet complete and from which we can infer that the following:

- Skin-microbiome profiling for human identification is a comparative analysis that requires the collection and analysis of skin samples and reference samples;
- Human microbiome identifiability is deeply connected with microbiome structure, personalization, and stability;
- To acquire forensic value, microbial identification should be reliable and reproducible, even after a large temporal interval;
- The huge quantity of data that can be extracted from a microbiome analysis has to be characterized and classified to obtain information which is not only useful for identification, but which is also admissible as forensic evidence in court.

## 5. Conclusions

In conclusion, the application in real forensic cases of microbiome-based analysis to achieve a reliable discrimination between individuals is a promising tool that is particularly useful when other identification techniques cannot provide useful information. Nevertheless, further in-depth studies would be necessary to verify the effective applicability of microbial analysis to human identification in real forensic settings.
